# Facing the threat: common yellowjacket wasps as indicators of heavy metal pollution

**DOI:** 10.1007/s11356-020-09107-2

**Published:** 2020-05-18

**Authors:** Oksana Skaldina, Robert Ciszek, Sirpa Peräniemi, Mikko Kolehmainen, Jouni Sorvari

**Affiliations:** 1 Department of Environmental and Biological Sciences, University of Eastern Finland, PO Box 1627, FI-70211 Kuopio, Finland; 2 A. I. Virtanen Institute for Molecular Sciences, University of Eastern Finland, PO Box 1627, FI-70211 Kuopio, Finland; 3 School of Pharmacy, University of Eastern Finland, PO Box 1627, FI-70211 Kuopio, Finland

**Keywords:** Bioindicator, Biomonitor, Biomarker, Colour trait, Heavy metals, Vespula vulgaris

## Abstract

We investigated the common wasp, *Vespula vulgaris* as a bioindicator and biomonitor of metals in the industrial area. Using traps, we collected 257 yellowjackets along a pollution gradient in the Harjavalta *Cu*-*Ni* smelter in Southwest Finland. Our method detected metal elements such as arsenic (*As*), cobalt (*Co*), copper (*Cu*), iron (*Fe*), nickel (*Ni*), lead (*Pb*), zinc (*Zn*), and mercury (*Hg*) in wasps*.* The data analyses revealed *V. vulgaris* can be a proper indicator for *As*, *Cd*, *Co*, *Cu*, *Ni,* and *Pb*, rather than for *Fe* and *Zn* contamination. Body burdens of *As*, *Cd*, *Co*, *Cu*, *Ni*, and *Pb* decreased with an increase in distance from smelter. Enrichment factor (EF) followed the pattern *Pb* ˃ *Cd* ˃ *As* ˃ *Co* ˃ *Cu* ˃ *Ni*. The highest bioaccumulation (BAF) values were revealed for *Cd* (5.9) and the lowest for *Pb* (0.1). Specially designed software (*WaspFacer*) allowed revealing body burdens of *As*, *Cd*, *Co*, *Cu*, *Ni*, and *Pb* to be associated with rather smaller than more asymmetric facial colour markings in yellowjackets. These results add to the body of literature on how heavy metal contaminants can have tangible phenotypic effects on insects and open future opportunities for using wasps as indicators of metal pollution.

**Electronic supplementary material:**

The online version of this article (10.1007/s11356-020-09107-2) contains supplementary material, which is available to authorized users.

## Introduction

In terrestrial environments, metals originate from a variety of sources such as geogenic, atmospheric, industrial, transport, domestic, agricultural, and pharmaceutical (Martin and Coughtrey 1982). The pathways for metals entering the soil include runoff from industrial sites and roads, metal-based pesticides, phosphate fertilizers, sewer sludge, and atmospheric particle deposition (Gall et al. 2015). Elements such as cobalt (*Co*), copper (*Cu*), iron (*Fe*), manganese (*Mn*), molybdenum (*Mo*), selenium (*Se*), nickel (*Ni*), and zinc (*Zn*) are essential in trace levels as micronutrients for plants, animals, and humans. However, their excessive uptake might cause a toxic effect on organisms (Bodgen and Klevay 2000). The other metals such as arsenic (*As*), cadmium (*Cd*), mercury (*Hg*), and lead (*Pb*) have no useful biological function and can have toxic effects even at low concentrations (He et al. 2005). Toxic heavy metals can remain in terrestrial environments for years, accumulating in food webs and threatening biota. The impact of accumulating pollution is of paramount concern in localized industrial areas, for example, in areas where metal smelting takes place (Alloway 2012). With this type of pollution, landscape heterogeneity, including agricultural and urban areas, affects the deposition of pollutants and modifies local environmental contamination (Fritsch et al. 2010). Although current levels of industrial heavy metal pollution have been decreasing in the European Union (Tóth et al. 2016; EEA 2018) and the USA (US EPA 2018), the opposite trend is apparent in China (Li et al. 2014), African countries (Yabe et al. 2010), and several other developing countries (Krishna and Govil 2005; Mohiuddin et al. 2011). Therefore, efficient, robust, and low-cost methods of monitoring metal pollution are urgently required. Bioindicators may be one such solution, with the concurrent benefits of raising awareness of the utility of biodiversity in environmental monitoring.

In terrestrial environments, several methods are currently used to monitor the rates of metal contamination. First, there are several chemical assessment methodologies, including (*i*) direct measurements or modelling of metal activities, (*ii*) metal extraction methods, and (*iii*) the application of semi-permeable devices for heavy metal analyses in soils (Peijnenburg et al. 2007). The second group of methods is related to microbial biomass and microbial activity (Yuangen et al. 2006). Finally, it is possible to assess environmental contamination by using organisms as bioindicators and biomonitors (Martin and Coughtrey 1982). An indicator can simply indicate the presence or absence of a factor, e.g. heavy metal in this case, while a monitor species can show a biological response to a chemical dosage (Markert et al. 1999). Considering biomonitors, a biomarker approach has been intensively adopted in risk assessment frameworks over the last 20 years, making it a valuable complement to ecotoxicity testing (Amiard-Triquet et al. 2013). A biomarker can be defined as a biochemical, cellular, physiological, morphological, or behavioural change which can be observed in body tissues or fluids or at the level of the whole organism that reveals the exposure or the effects of one or more chemical pollutants (Depledge 1994). The major benefit of biomarkers is that they are sensitive early warning tools to assess environmental health.

Within terrestrial ecosystems, several plants and parts of the plants are used for biological monitoring purposes. The most frequent are mosses (Berg and Steinnes 1997), lichens (Bargagli 2016), trees (Sawidis et al. 2011), and bark from trees and shrubs (El-Hasan et al. 2002), as well as leaves and conifer needles from trees and shrubs (Aksoy and Öztürk 1997; Pietrzykowski et al. 2014). Although birds (Costa et al. 2012) and small mammals (Talmage and Walton 1991) are used as bioindicators and biomonitors, terrestrial invertebrates have several benefits. They are abundant organisms, with growth rates and a population turnover midway between microorganisms and higher plants and animals (Hodkinson and Jackson 2005). Invertebrates are usually in close contact with contaminants in the soil. Measurements of heavy metals from soil samples revealed that metal concentrations could be highly spatially heterogeneous (Salminen and Haimi 1999). Therefore, invertebrate bioindicators might better reveal the environmental quality than soil samples. There are significant differences in the metal bioaccumulation of various invertebrate taxa: high in the Isopoda and low in the Coleoptera (Heikens et al. 2001). For some taxa, the data is limited or not presented. Although a diverse range of insects has been studied regarding its usefulness in bioindication and biomonitoring (Heikens et al. 2001; Nummelin et al. 2007), only limited information exists for the social wasps (Hymenoptera, Vespidae) (but see Kowalczyk and Watala 1989; Urbini et al. 2006; Polidori et al. 2018). Recently, it has been stressed that metal contamination can increase disease susceptibility in social insects (Feldhaar and Otti 2020). Therefore, such studies possess wider ecological implications than just bioindication.

Here, we addressed two questions. First, we aimed to determine whether *Vespula* yellowjacket wasps accumulate local industry-originated heavy metals in their bodies and thus whether they are suitable environmental bioindicators for industrial heavy metal contamination. We attempted to achieve this aim by collecting samples across a pollution gradient near a *Cu*-*Ni* smelting area in Finland, and by conducting further analyses of individual metal levels in wasp bodies using inductively coupled plasma mass spectrometry (ICP-MS).

In a range of species, the colouration has been shown as a metal-sensitive trait, reflecting the accumulation of metals in organisms and indicating environmental metal contamination (Pérez et al. 2010; Lifshitz and St Clair 2016; Skaldina and Sorvari 2017; Skaldina et al. 2018). Therefore, in the second aim, we intended to identify any colour traits in faces of yellowjacket wasps that could be associated with industrial metal contamination, suggesting the ways these traits can assist current biomonitoring programmes. To do this, we assessed facial melanin markings with specially designed software—the *WaspFacer*.

## Materials and methods

### Study species

**Fig. 1:**
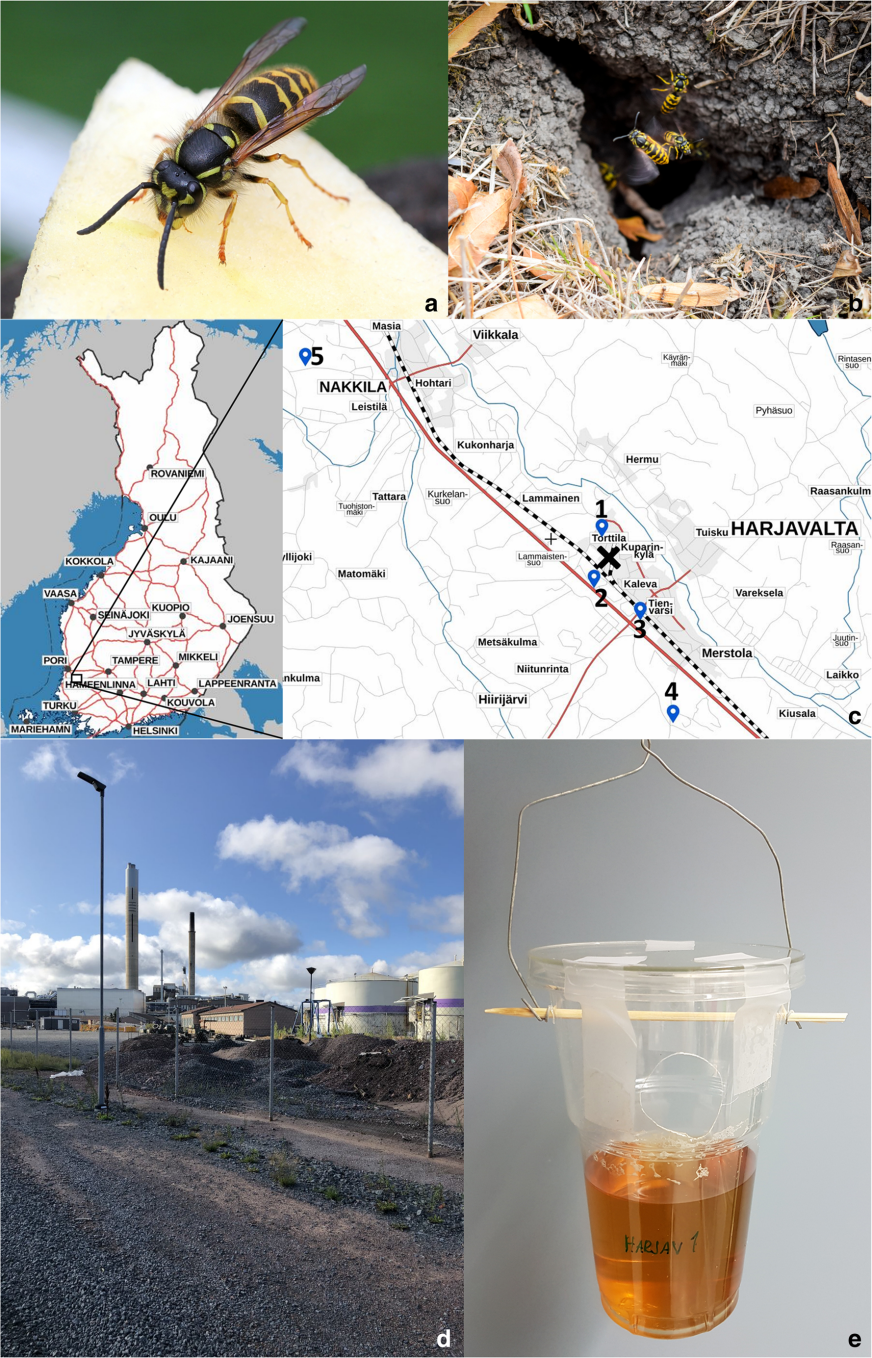
Study species (A and B) and sampling method, study area (C and D), and a beer trap (E). The study area names in this study: 1: Torttila, 2: Gate, 3: Nummi, 4: Hiite, 5: Nakkila. Photographs A and B were purchased with the standard licence from Shutterstock, © Daniel Prudec. All rights reserved

The common yellowjacket wasp *Vespula vulgaris* (Hymenoptera, Vespidae) (Fig. 1A) is a eusocial species with an annual life cycle. It is both native and invasive to the Holarctic region (Dvořák 2007), and invasive in Australia, Tasmania, New Zealand, Hawaii, and South America (Mathews et al. 2000; CABI 2019; Lester and Beggs 2019). Yellowjackets are highly flexible in their nesting and foraging habits and consequently occur in both natural and anthropogenic landscapes. *V. vulgaris* build carton nests in mammal-made holes in the ground, hollow trees, or in anthropogenic cavities (e.g. attics, disused chimneys, sheds) (Fig. 1B). Therefore, the wasp colonies are usually in close proximity to the soil. *V. vulgaris* is a generalist predator of both ground-dwelling and flying arthropods (Richter 2000). To feed brood, it preys on Diptera (flies), Lepidoptera (moths), Araneae (spiders) (Harris and Oliver 1993), and ants (Formicidae) (Harris 1991). The adult wasps widely consume carbohydrates such as fruit, flower nectar, and aphid honeydew to maintain their energy resources, and they collect wood pulp for the nest construction (Greene 1991). Nesting and feeding preferences of this species, therefore, predispose both adults and their offspring to the uptake of heavy metals from different strata of the terrestrial environment and considering these wasps as bioindicators and biomonitors of metal pollution.

### Study area

We sampled wasps at the Harjavalta town and its vicinities (61° 19′ N, 22° 9′ E) in South-Western Finland in August 2014. In that area, the dominant tree was Scots pine, *Pinus sylvestris* L., and the dominant forest scrub species varied from *Vaccinium* to *Calluna*; the soil type was ferric podzol (Derome and Lindroos 1998). Harjavalta is the largest heavy metal polluted zone in Finland, but the area of forest death there is still less than 1 km^2^ (Salemaa et al. 2001). The local source of pollution is a copper-nickel smelter, emitting heavy metals and sulphur during the last 50 years. Besides main metals such as *Cu* and *Ni*, emissions from the smelter contain arsenic *As*, *Cd*, *Pb*, *Fe*, *Hg*, and *Zn* (HELCOM 2002). In addition, *Co* is a common by-product of the *Ni*-mining process; for example, a typical nickel slug contains 0.1% of *Co* (Riekkola-Vanhanen 1999). Thus, some cobalt pollution might be also depicted in the studied area.

At Harjavalta, the emission of heavy metals started to decrease from the beginning of 1990s; a steep decrease was noticed in *Ni* concentrations, but less marked in *Cu*. That decrease was primarily due to novel technical modifications to the smelter (e.g. installations of new filters) and the construction of a taller smokestack (Berglund et al. 2015). Current levels of pollution in Harjavalta do not pose toxicological risks for humans and wildlife (Eeva et al. 2018). Anyway, due to existing and long-term industrial pollution, in the vicinity of a smelter soil still contain elevated metal concentrations (Berglund et al. 2015). A clear decreasing gradient for soil concentrations of metals such as *Cd*, *Cu*, *Fe*, *Ni*, *Pb*, and *Zn* was revealed at the distances 0.5, 2, 4 and 8 km from the smelting plant (Derome and Lindroos 1998). The overall ecosystems’ conditions follow a similar gradient of distance. In the immediate proximity to plant (˂ 1 km), heavy metal pollution has variable detectable effects on organisms’ survival, reproduction, and biodiversity (Kiikkilä 2003). At about 2 km from the smelter, clear signs of nutrient cycling disturbance are still visible. At 4-km distance, there are noticeable changes in species composition, although the effects of pollution are not so acute anymore. At 8 km, the overall ecosystem can be classified as undisturbed with only slight changes in the understory vegetation. For this reason, we carried out a sampling campaign at five study sights across the pollution gradient at a distance ranged from 0.86 to 10.66 km (Fig. 1C) from the smelting plant (Fig. 1D).

### Sample collection and pre-processing

A total of 257 wasps were collected at the area across the pollution gradient (≤ 10.66 km to smelter) using a beer-trapping method (Fig. 1D) (Sorvari 2013). Altogether 20 beer traps were left out for 1-week period in early August 2014. GPS coordinates were taken to identify the location of each trap. A detailed description of the locations’ distance to the smelter, number of traps, and wasps per location is given in the supplementary table (Table S1A). The distance between the nearest-neighbour traps ranged from 20 to 50 m, and the maximum distance between traps within the same location was 200 m. After remaining for 1 week in the field, the traps were emptied, and wasps from each trap were placed in plastic tubes in a freezer at a temperature of − 20 °C. As shown by Braun et al. (2009), short periods (˂ 2 weeks) of insects’ presence in trapping fluids did not result in metal leaching or cross-contamination of samples. Also, no effects of a short stay in beer have been previously observed to affect wasps’ colouration. Later, we identified *V. vulgaris* specimens using the identification key by Dvořák and Roberts (2006).

### Digital photographing and image pre-processing

The wasp faces were photographed using a Nikon DS-Fi1 microscope camera (5-megapixel CCD) and DS Camera Control Unit attached to an Olympus SZX9 trinocular microscope with a magnification of × 16. The photographs were taken with NIS-Elements BR imaging software version 3.2 in a windowless room under stable light conditions (058 PAR) and saved as 8-bit RGB images. One person carried out photographic imaging over 2 days in September 2017. To obtain good-quality photographs in a stable position, and to avoid additional metal contamination, we fixed each wasp in small plastic tubes. We also carried out all manipulations with nitrile examination gloves and used only plastic or wooden materials to perform the manipulations. Digital photographs of the wasps’ thoraxes were additionally taken for the size measurements.

### Photographic analyses: WaspFacer

**Fig. 2:**
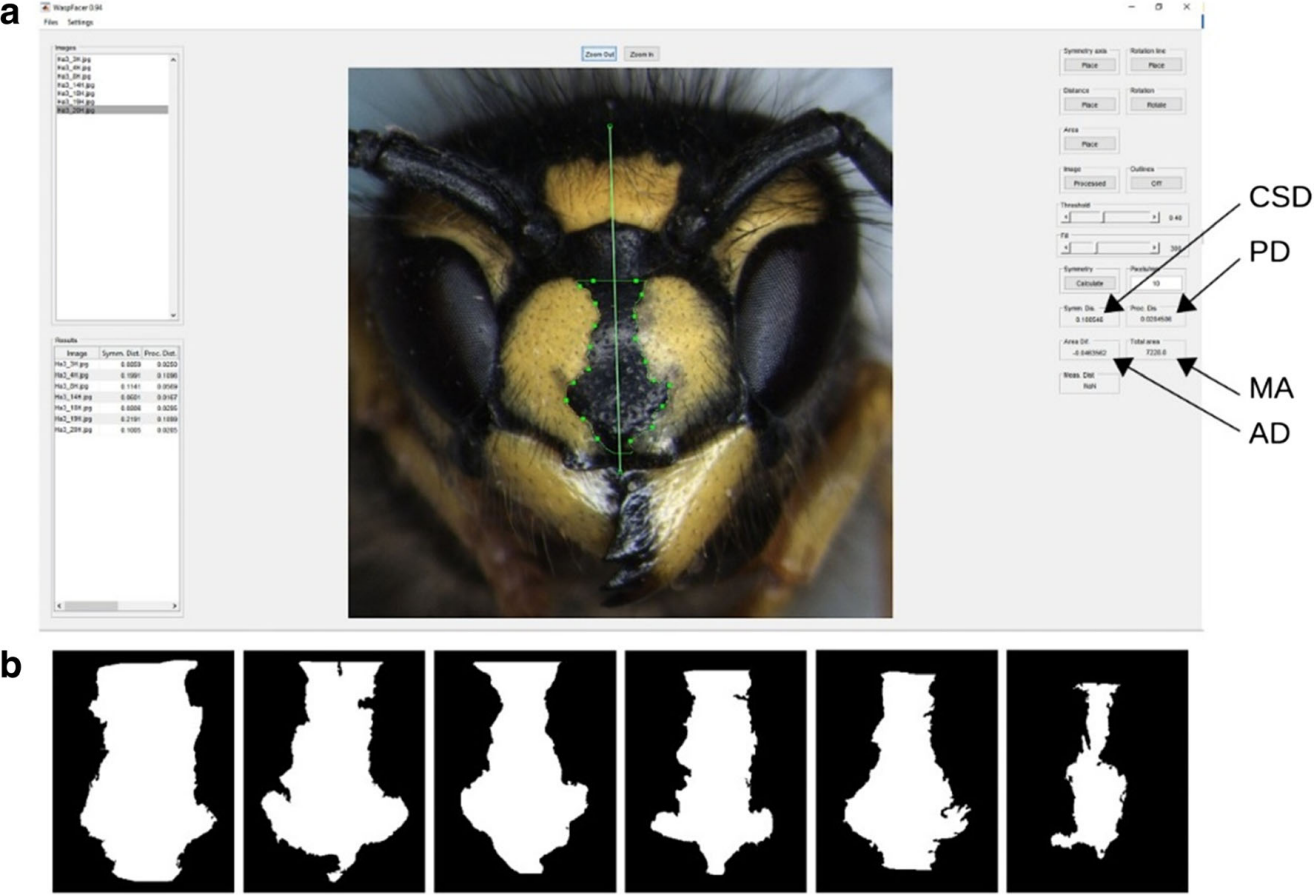
A view of the WaspFacer and facial colour trait analyses of *Vespula vulgaris*. (A) The user interface with a single analysed image. The vertical green line denotes the symmetry axis defined by the user. Scored parameters are continuous symmetry distance (CSD) same as a continuous symmetry measure (CSM); Procrustes distance (PD); area difference (AD); melanisation area (MA). (B) A set of exported bitmap images of the segmented facial markings

To ease the analysis of the different parameters of the facial colour markings of the wasps, we developed a new open-source software tool—*WaspFacer* (Fig. 2). The software was implemented using MATLAB 2018b and can be downloaded from https://rciszek.github.io/WaspFacer. The purpose of the software was to enable a fast and semi-automatic method for the analysis of colour traits, which facilitated the processing of larger sample sets to assist research and biomonitoring. *WaspFacer* enabled the fast and convenient assessment of several parameters for each anchor-shaped facial colour marking (Fig. 2A). These parameters were (*i*) a continuous symmetry distance (CSD) here and after a continuous symmetry measure (CSM), (*ii*) the Procrustes distance (PD), (*iii*) the area difference (AD), and (*iv*) the area of melanisation (MA). A detailed description of the program and the scored parameters and reasoning for selecting facial colour parameters is available at the supplement S2.

### Size and weight measurements

The size measurements included the head width (HW) and thorax width (TW) in millimetres (mm) and were taken from the digital photographs using the *WaspFacer* and ImageJ software. The HW was measured as the maximum interocular distance, which was the distance between the concavities of the wasps’ compound eyes (Karsai and Hunt 2002) and the TW was measured as an intertegular distance, which is the distance between the bases of the right and left wings—the tegulas. This distance had been confirmed as a reliable estimator for the individual body size in Aculeata (Greenleaf et al. 2007; Karsai and Hunt 2002). Measurements of individual dry body mass are further described in the next subsection. Dry body mass also better predicts body size due to reduced variation from water content (Ohl and Thiele 2007).

### Heavy metal analysis

The following metal elements were quantified from individual yellowjacket samples: arsenic (*As*), cadmium (*Cd*), cobalt (*Co*), copper (*Cu*), iron (*Fe*), mercury (*Hg*), nickel (*Ni*), lead (*Pb*), and zink (*Zn*) using inductively coupled plasma mass-spectrometer (ICP-MS). The calibration of the instrument was done using a certified solution standard (TraceCERT Periodic Table Mix 1 and Mercury standards for ICP, Sigma-Aldrich). Element isotopes without known spectral interferences were preferentially selected for analysis. A triple-quadrupole reaction system was used to remove polyatomic interferences for elements that do not have abundant, interference-free isotopes. The reaction system was operated in collision mode with kinetic energy discrimination (KED), using helium as the cell gas (3.7 mL min^−1^). A peristaltic pump and nebuliser were used for sample injection. Three internal standards, litium-6, rhodium-103, and uranium-238, were mixed online with the samples to compensate for matrix effects and instrument drift. Method blanks and certified standard reference material NIST® SRM® 1577b Bovine Liver (Sigma-Aldrich) were measured in addition to the unknown samples for each batch of analysis to check for contamination and to confirm the accuracy of the analysis batch. After the photographs were taken, all the wasps were dried in an oven Termarks 8000 for 48 h at 60 °C and cooled down for 48 h in a desiccator at 20 °C to prevent additional moisture. Then we measured the individual dry body mass (M_b_ or d.w.) to the nearest 0.001 mg using a Metler Toledo MX5 analytical balance. This was needed because an analysis for heavy metal levels was performed for each wasp individually. Wasp samples (0.015–0.020 g) were dissolved with a microwave digestion system using the MARS™ 6 iWave instrument (CEM Corporation, USA) and the Animal Tissue method in 8 ml of HNO_3_ (TraceMetal™ grade, Fisher Scientific) using MARSXpress teflon digestion vessels. Simultaneous performance of analytical blanks and certified reference material confirmed the method accuracy was within acceptable limits. After digestion, the samples were diluted to 20 mL with de-ionized water (USF Elga Maxima). The determination of metal concentrations was performed with NexION 350D ICP-MS (PerkinElmer, USA) equipped with ESI prepFAST autosampler (Elemental Scientific, USA). The detection limit for most of the elements was approximately 0.0001 μg/g. The range of detected metals in wasps’ tissues is presented in microgrammes per gramme on a dry weight.

### Statistics and index calculation

Enrichment factor (EF) was calculated as suggested by Nieminen et al. (2002) and Eeva et al. (2018). Mean element concentrations in the highly polluted area (≤ 2 km) were divided by mean element concentrations in the less polluted area (≥ 4 km) in a sample type (wasps: μg/g, d.w.). Bioaccumulation factor (BAF) was calculated as the average metal concentration in wasps (μg/g, d.w.) divided by average topsoil concentrations (μg/g, d.w.) in the highly exposed zone (˂ 2 km). We used soil concentration for the elements such as *As*, *Cd*, *Co*, *Cu*, *Ni*, and *Pb* reported for the distance 0.9 km from the smelter by Ruiz et al. (2017). BAF indexes for *Fe*, *Co*, and *Zn* were not calculated as no appropriate soil references were found. Soil *Zn* concentrations provided by Kiikkilä (2003) were 17 years old, therefore cannot be considered for the research purposes in the current paper.

Distribution of heavy metals was normalised by logarithmic transformation except for EF and BAF indexes. We used a Pearson coefficient to reveal the correlation between levels of heavy metals and the distance from the smelting plant, for revealing correlation between metal values, and for revealing correlations between several metal elements and between phenotypic characteristics. A principal component analysis (procedure FACTOR in SAS) was done for eight intercorrelated heavy metal elements using Varimax rotation and Kaiser normalization. Analyses of the effects of logarithmical distance on the PCs were done using linear mixed models (LMM, the MIXED procedure in SAS); models’ residuals were normally distributed.

Analyses of the body size differences, and several colour traits and metal levels were performed using linear mixed models (LMM, the MIXED procedure in SAS). Since the average foraging distance of *V. vulgaris* is 80 m (Richter 2000), and the traps were placed in the field at a distance of 20–50 m, trap identity was not used as a random factor in the models. However, the sampling location was used as a random factor in analyses on the association between metal levels and distance to the smelter complex. All pairwise comparisons were made using the Tukey-Kramer test. All mean values are presented with 95% confidence intervals. Phenotypic characteristics were analysed in model that requires normality of model residuals, which was fulfilled. The analyses were carried out using the SAS statistical software, version 9.4 (SAS Institute Inc.).

## Results

### Heavy metal load

The ICP-MS method revealed the presence of metal elements and their body burdens in yellowjackets (μg/g, d.w.) such as *As*—from 0.7 to 40.7; cobalt *Co*—from 0.1 to 841, *Cu*—from 28 to 289; *Cd*—from 0.1 to 19.9; *Fe—*from 143 to 8316; *Ni—*from 0.7 to 5502; *Pb—*from 0.1 to 18.3; *Zn—*from 50 to 13,381. Because *Hg* was found in wasps only from two of the closest locations to the smelter (Torttila and Gate), we did not perform further analyses for that element. Mean values for body burdens of these elements, EF and BAF values, are presented in Table 1. The highest values for the EF were revealed for *Pb* (7.3), *Cd* (5.4), *As* (4.7), *Co* (2.5), *Cu* (2.1), and *Ni* (2); lower values were found for *Fe* (1.7) and *Zn* (1.1). The highest BAF values were revealed for *Cd* (5.9) and the lowest for *Pb* (0.1).

**Table 1:** Mean values (mean ± SD) for the individual yellowjackets’ body burdens (μg/g, d.w.) of the main heavy metal pollutants in high (≤ 2 km) and lower (˃ 4 km) exposure zones, enrichment factor (EF), and bioaccumulation factor (BAF), shown for soil→wasps transfer routes for the polluted areas (*n* = 257 wasps)

Element	Higher exposure area	Lower exposure area	EF	BAF
*As*	7.94 ± 5.36	1.69 ± 1.08	4.7	1
*Cd*	2.35 ± 2.91	0.44 ± 0.43	5.4	5.9
*Co*	2.27 ± 6.65	0.92 ± 2.93	2.5	–
*Cu*	98.7 ± 51.3	47.4 ± 12.5	2.1	0.4
*Fe*	398 ± 909	244 ± 358	1.7	–
*Ni*	75.6 ± 429	38.3 ± 196	2	1.5
*Pb*	2.25 ± 2.36	0.31 ± 0.22	7.3	0.1
*Zn*	787 ± 1385	707 ± 618	1.1	–

The levels of *As*, *Cd*, *Co*, *Cu*, *Ni*, and *Pb* negatively correlated with the logarithmic distance from the smelting plant. For *Zn*, the correlation was weakly positive, yet significant; weak negative, but marginally non-significant correlation was revealed for *Fe* (Table S1 B). All elements had significant intercorrelated values, except *Zn*, which correlated only with *Co* and weakly with *Fe* (Table S1 C).

**Fig. 3:**
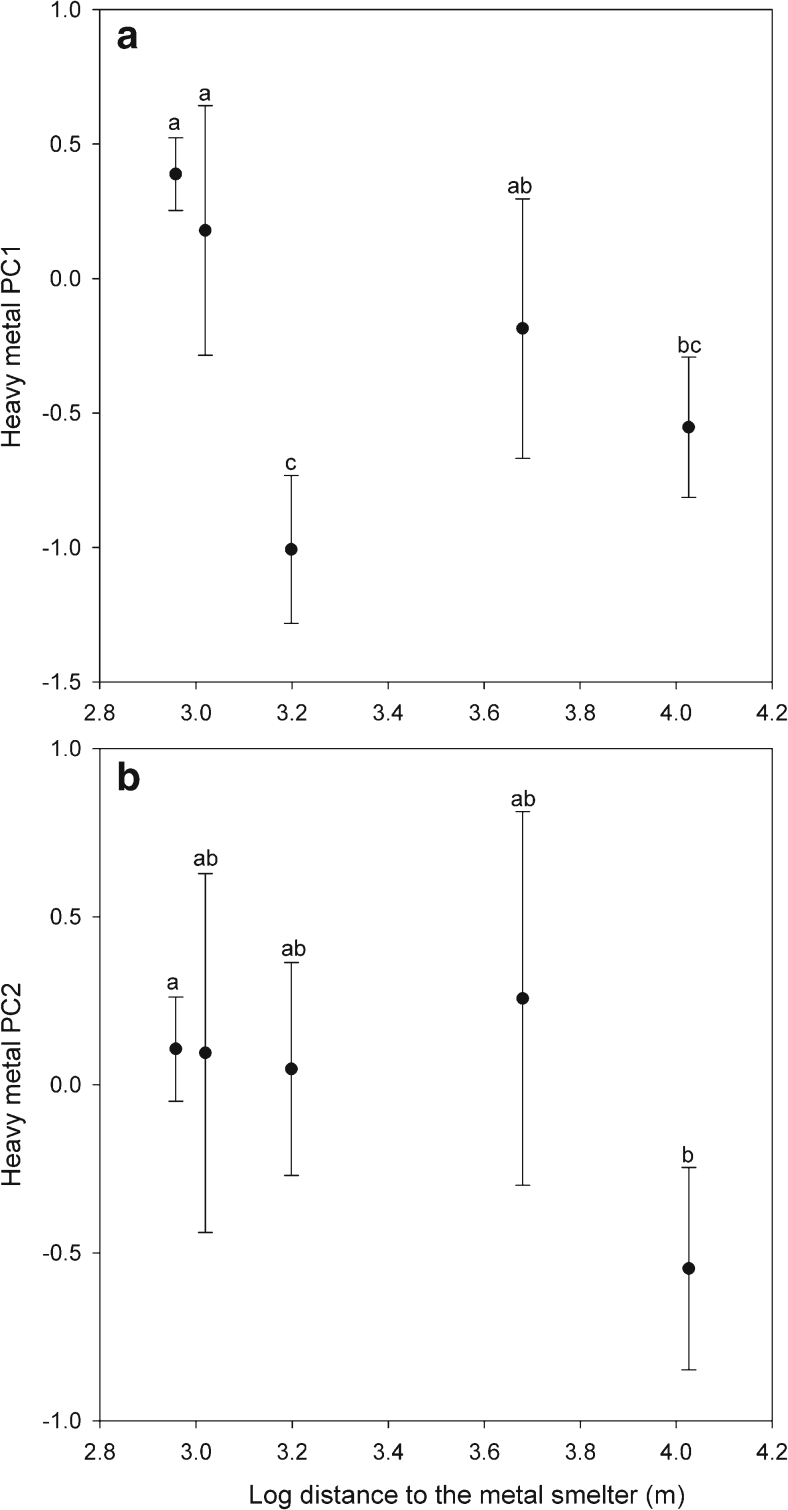
The association between (A) PC1 (*As*, *Cd*, *Cu*, and *Pb*) and (B) PC2 (*Ni* and *Co*) and the logarithmic distance of the wasps’ traps (*n* = 20) to the Harjavalta *Cu*-*Ni* smelting plant. Symbols for the sites from left to right: Torttila, Gate, Nummi, Hiite, and Nakkila. The different letters above the symbols indicate significant pairwise differences between the areas (Tukey’s test *p* ˂ 0.05)

A principal component analysis (PC) for metal concentrations from individual wasp samples revealed two PCs with eigenvalues over one (PC1: 4.53 and PC2: 1.22). PC1 explained 56.59% of the variance and was associated with *As*, *Cd*, *Cu*, and *Pb*; PC2 explained 15.19% and was associated mainly with *Ni* and *Co* (Table 2). PC1 (*As*, *Cd*, *Cu*, and *Pb*) decreased with an increasing logarithmic distance from the smelting plant (*F*_1,255_ = 31.00, *p* < 0.0001; Fig. 3A). Similar negative association was found for PC2 (*F*_1,255_ = 11.6, *p* = 0.0008; Fig. 3B). In addition, both PC1 and PC2 values differed significantly between the studied areas (PC1: *F*_4,252_ = 25.7; *p* ˂ 0.0001: Fig. 3A; PC2: *F*_4,252_ = 3.92, *p* = 0.0042 Fig. 3B).

**Table 2:** Correlations between logarithmically transformed metal eigenvalues and two principal components (PC1 andPC2)

	Rotated factor pattern	
	PC1	PC2
*Fe*	0.081	0.225
*Co*	0.338	0.498
*Ni*	0.165	0.893
*Cu*	0.401	0.231
*Zn*	− 0.013	− 0.017
*As*	0.483	0.276
*Cd*	0.869	0.176
*Pb*	0.303	0.224

### Colour traits and heavy metals

**Fig. 4:**
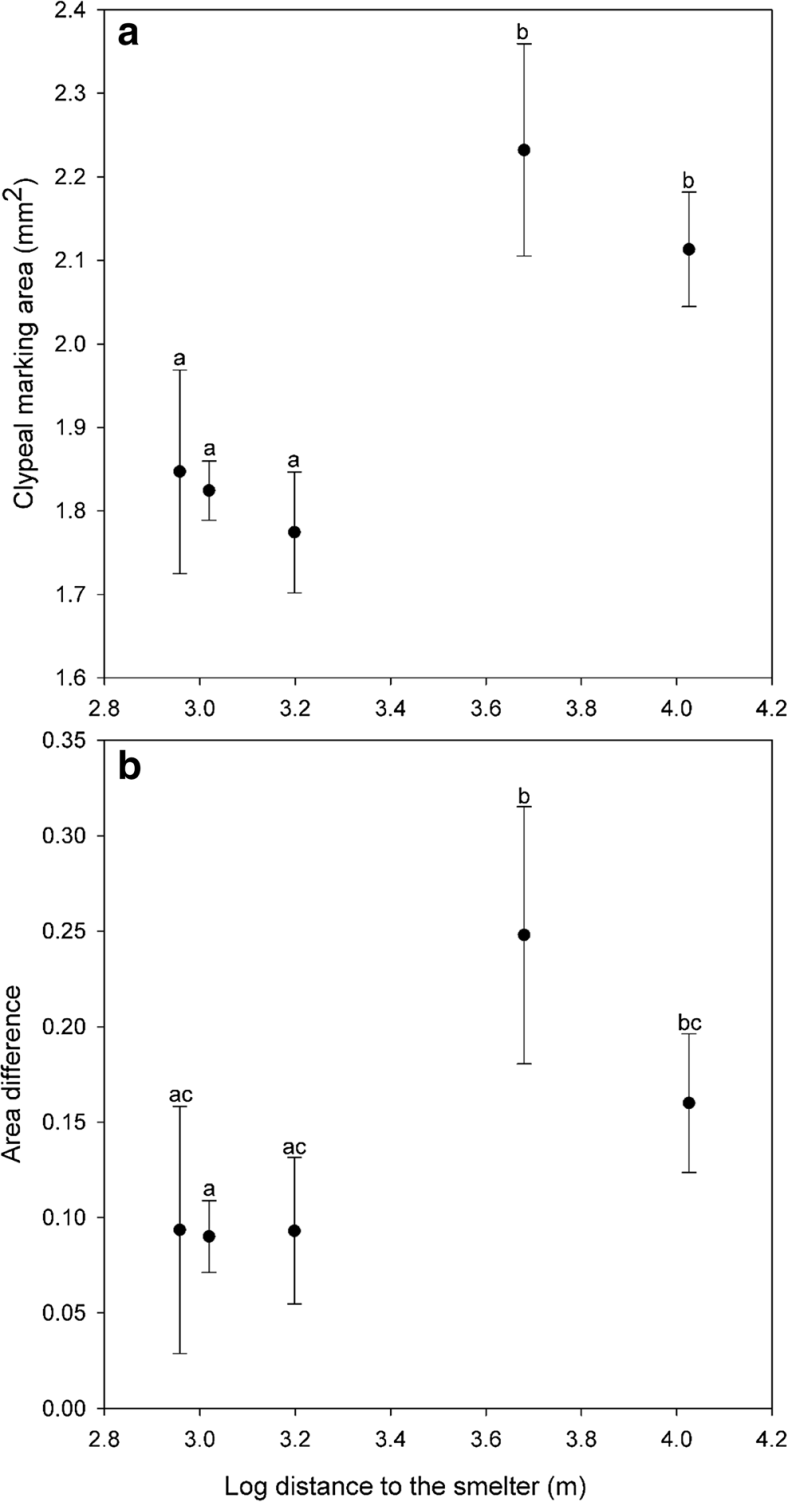
(A) The difference in the area of clypeal melanisation (MA in mm^2^) and (B) distinctions in (non-directional left/right differences) in the area of clypeal melanisation (AD in mm^2^) of *V. vulgaris*, collected along the pollution gradient (logarithmical distance to the smelting plant). The symbols of the sites from left to right: Torttila, Gate, Nummi, Hiite, and Nakkila. Different letters above the symbols indicate significant pairwise differences between areas (Tukey’s test *p* ˂ 0.05)

To check if facial colour traits in yellowjackets can reflect heavy metal pollution, we analysed the traits such as MA, AD, PD, and CSM of the anchor-shaped melanin marking and the interrelationship of those traits with the logarithmic distance to smelter and heavy metal body burden. Closer to smelter (˂ 2 km) and further away (˃ 4 km) *V. vulgaris* differed in the MA (*F*_4,252_ = 23.2, *p* < 0.0001; Fig. 4A; Table 3) and AD (*F*_4,252_ = 7.2, *p* < 0.0001; Fig. 4B). However, no significant differences were revealed for CSM (*F*_4,252_ = 1.11, *p* = 0.353) and PD (*F*_4,252_ = 0.79, *p* = 0.531). These findings suggested that closer to smelter wasps had significantly smaller area of facial colour markings and smaller difference between right and left part of the marking. MA and AD decreased with increasing levels of *Cu*, *As*, *Cd*, and *Pb*, and increase of *Ni* and *Co* decreased all parameters (MA, AD, PD, CSM) except *Co* for AD (Table S1 D).

**Table 3:** Estimated marginal means (mean ± SD) for the head width (HW), thorax width (TW), body mass (M_b_), and melanisation area of the clypeus (MA) in yellowjackets (*n* = 257) from high exposure areas (Torttila, Gate, Nummi) and lower exposure (Hiite and Nakkila) areas

	HW (mm)	TW (mm)	M_b_ (mg)	MA (mm^2^)
Torttila	2.64 ± 0.01	2.5 ± 0.02	16.21 ± 0.31	1.85 ± 0.12
Gate	2.62 ± 0.05	2.5 ± 0.07	16.66 ± 1.05	1.82 ± 0.04
Nummi	2.61 ± 0.03	2.4 ± 0.04	17 ± 0.62	1.77 ± 0.07
Hiite	2.53 ± 0.05	2.4 ± 0.07	17.6 ± 1.09	2.23 ± 0.13
Nakkila	2.57 ± 0.03	2.4 ± 0.04	16.87 ± 0.05	2.11 ± 0.07

Further, we checked if melanin facial colour traits of yellowjackets were associated with body size parameters, measured as (*i*) head width (HW), (*ii*) thorax width (TW), and (*iii*) dry body mass (M_b_). All body size parameters were weakly correlated between each other (M_b_ × HW: *r* = 0.26, *p* ˂ 0.0001; M_b_ × TW: *r* = 0.24, *p* ˂ 0.0001; HW × TW: *r* = 0.27, *p* ˂ 0.0001). A between-area comparison using linear mixed model analyses revealed that the yellowjackets significantly differed in HW: *F*_4,252_ = 8.72, *p* = 0.0001; TW: *F*_4,252_ = 4.9, *p* = 0.0008 and M_b_ (*F*_4,252_ = 2.87, *p* = 0.024) along the pollution gradient at the five study sites (Table 3).

## Discussion

### Common wasps as bioindicators of industrial Cu-Ni pollution

Our results confirmed that yellowjackets indicate *As*, *Cd*, *Co, Cu*, *Ni*, and *Pb* industrial contamination. Right next to the smelting plant and at the distance up to 2 km away, they accumulated metal elements in amounts at least two times higher, compared to reference areas. Although *Fe* and *Zn* were also detected in wasps, there was almost no difference in those between zones. Predatory insects differ in their capability for metal accumulation. Thus, waterstriders (Hemiptera, Gerridae) and antlions (Neuroptera, Myrmeleontidae) were shown as good bioindicators for *Fe* and dragonfly (Odonata) larvae as bioindicators for *Zn* (Nummelin et al. 2007). However, our data did not reveal *V. vulgaris* as a bioindicator for *Fe* and *Zn*, although in the environments with predominated *Fe* or *Zn* exposure, the situation might be different.

The revealed order of the metal content decrease in wasps was consistent with that reported by Migula et al. (2013) for the spiders (Araneae) but differed from the grasshoppers (Orthoptera) (Karadjova and Markova 2014). Both spiders and wasps are top-level predators, in which bioaccumulation might be dependent on their “hunting behaviour, chemical load in prey, gender, age, season and an intensity of exposure to metals” (Migula et al. 2013). Compared to other elements, essential metals such as *Zn*, *Fe*, and *Cu* reached the highest concentrations in wasps. Insects normally need these metals for energy production, nutrient metabolism, and gene regulation; however, their excess can disrupt gene expression, cause enzyme malfunction, and lead to oxidative stress (Merritt and Bewick 2017). For the model insect organism *Chironomus ramosus* (Diptera, Chironomidae), median lethal concentrations (LC_50_) for copper (CuSO_4_ × 24 h) exposure were determined as 3280 μg/g (acute toxicity) and 1.8 μg/g after 21-day exposure (chronic toxicity) (Majumdar and Gupta 2012). However, for the yellowjackets, acute and chronic toxicity doses for *Cu* and other essential metals (e.g. *Fe*, *Mn*, *Zn*) have not been yet revealed.

Several previous studies provide a clear proof that in Harjavalta, there is a decreasing gradient of soil-deposited metals (Derome and Lindroos 1998; Kiikkilä 2003; Ruiz et al. 2017). We revealed that when assessing distance as a gradient, body burdens for the elements such as *As*, *Cu*, *Co*, *Cd*, *Fe*, *Ni*, and *Pd* in wasps were decreasing, but not the concentrations of *Zn*. Del Toro et al. (2010) previously got similar results for the granivorous ant *Pogonomyrmex rugosus* (Hymenoptera, Myrmicinae), revealing a decrease of these elements (*As*, *Cu*, *Cd*, *Pd*) with distance from the *Cu*-*Pb* smelter in Chihuahuan Desert (Mexico, USA). Similarly, Eeva et al. (2004) identified decreasing gradient for *As*, *Cu*, *Pd*, and for *Ni* concentrations in red wood ants (*Formica* s. str) in Harjavalta. Contrary, any decline in the concentrations of *Cu* and *Pb* in several predatory insects such as waterstriders, antlions, and dragonflies was found by Nummelin et al. (2007) in the vicinity of the Koverhar iron-steel factory. Given examples might confirm that some insect species, as a common wasp in our case, might not simply indicate the presence or absence of a contaminant in the environment but also reflect the gradient for its decrease.

When comparing internal body concentrations with soil concentrations in different invertebrates, it has been shown that the slope for metal accumulation patterns (BAFs) decreased in the order: *Pb ˃ Cd* ˃ *Cu* (Heikens et al. 2001). However, similarly to our results (the highest BAF values were revealed for *Cd*; lower for *Pb* and *Cu* in wasps), the same tendency existed for the ants (BAFs: seeds→ants) (Del Toro et al. 2010). For *Cd*, both EF and BAF values were high in our samples, which can be the evidence for the dependence of *Cd* accumulation in wasps on soil metal concentration in the environment. Although EF for *Pb* in *V. vulgaris* was even higher, its BAF value was the lowest. This finding might indicate the predominant role of the other than soil exposure source (might be contaminated food) of *Pb* accumulation in common wasps. Previous studies on heavy metals in wasps confirmed the high capability of these insects for *Cd* (Kowalczyk and Watala 1989) and *Pb* accumulation (Urbini et al. 2006; Polidori et al. 2018). Here, we for the first time revealed the capability of wasps for *As* and *Cu* accumulation*. As* accumulation might depend on the subgroup of invertebrates (Moriarty et al. 2009) and has been previously shown to be high in ants (Eeva et al. 2004; Moriarty et al. 2009).

In addition, aquatic insects are important vectors for the transfer of arsenic from lakes and streams to terrestrial predators (Mogren et al. 2013). Previously, Tarvainen et al. (1997) showed that, in the industrialized southwest region of Finland, there were elevated concentrations of *As* in surface waters, and its elevated levels correlated with high levels of *Cu*. In Harjavalta, there is a river Kokemäenjoki, located at less than 2 km to the smelter. The aquatic insects, inhabiting that river, might be an important source of metal exposure to wasps. Wasps might take up metals mostly with their food (Hopkin 1989). They have diverse diet, including carbohydrates such as honeydew from insects’ excretions or flower nectar (Spradbery 1973), protein-based food—arthropods from the orders Hemiptera, Lepidoptera (Harris 1991), Diptera, Hymenoptera, and Araneae (Harris and Oliver, 1993) or lipid-rich seed appendages—elaiosomes (Jules 1996). Therefore, in terrestrial environments, there are many sources for wasps’ exposure to heavy metals, which might be considered in future research.

### *V. vulgaris* as a biomonitor for heavy metal pollution

We revealed that near to Harjavalta *Cu*-*Ni* smelter, common wasps possessed decreased areas of their facial melanin-based colour traits. That was associated with elements such as *As*, *Cd*, *Co*, *Cu*, *Ni*, and *Pb*. Pollution did not apparently result in the increased asymmetry of that facial colour markings; however, some association between *Ni* and *Co* with facial pattern asymmetry was found. The finding that *V. vulgaris* workers had smaller areas of melanisation on their faces closer to the metal smelting plant was consistent with our previous results. Ants (*Formica lugubris*) had less melanised heads in industrially polluted environments (Skaldina et al. 2018). Describing the interrelation between metals and melanin pigments, McGraw (2003) suggested that melanin could bind metals, which might result in increased melanized areas. However, this may be dependent on the species-specific metal deposition areas, which in insects are commonly midgut, fat body, Malpighian tubules, and sometimes exocuticle (Ballan-Dufrançais 2002; Lukáň 2009). Insects use the following mechanisms to tolerate heavy metal pollution: (*i*) avoidance of the exposure source, (*ii*) limited uptake of contaminants, (*iii*) immobilization and (*iv*) elimination of metals, and (*v*) metal excretion (Heikens 2001; Grześ 2010; Migula et al. 2013). Immobilization of metals occurs via encapsulation, which in insects requires melanin pigments (Nakhleh et al. 2017). Then in polluted environments, there might be a cost to produce melanin-based colour traits. Metal encapsulation in *V. vulgaris* was already shown by Polidori et al. (2018). Therefore, decreased areas of facial melanin colour patterns in common wasps from polluted environments might be explained by a trade-off for melanin utilization needed for metals detoxification and encapsulation over colour pattern development.

The impact of metals on terrestrial ecosystems and the distinct species depends on the metal bioavailability and bio-accessibility and also on the species’ reaction (Luoma and Rainbow 2005). Previously, it has been shown that heavy metals can have diverse ecotoxicological effects on insects. Intensive *Pb* exposure resulted in smaller morphological traits in aphids (Görür, 2006) and in decreased body mass in beetles (Osman et al. 2015). *Cd* caused metabolic disorders in ants (Migula et al. 1993); in combination with *Hg*, it provoked increased mortality rates (Migula et al. 1997). Consistently with the other studies (Osman et al. 2015; Zhan et al. 2017), including our own (Skaldina et al. 2018), insects may suffer a reduced body mass in response to heavy metal pollution. Combined metals might affect insects’ development in various ways. For example, elevated *Zn* increased the growth rate, while excess *Cd* has been seen to reduce the survival of aphids (Stolpe and Müller 2016). *Cu*, *Fe*, and *Zn* might act as micronutrients (Bodgen and Klevay 2000), resulting in body size increase. However, as previously revealed for ground beetles (Osman et al. 2015) and wild bees (Moroń et al. 2014), *Cd* might be specifically responsible for a body mass decrease. Recently, body size was shown as a predictor of mortality for several aquatic insects (Cadmus et al. 2020). More studies are needed to reveal if there are similar trends for the terrestrial insects, especially for predatory wasps.

## Conclusions

The common wasp *V. vulgaris* can be used as a bioindicator for revealing metals such as *As*, *Cd*, *Co*, *Cu*, *Ni*, and *Pb*, rather than for *Fe* and *Zn*. However, this might change in the environments with the other metal exposure sources. Our results confirmed that concentrations of metals (*As*, *Cd*, *Co*, *Cu*, *Ni*, and *Pb*) gradually decreased with the distance from the smelter and had the highest enrichment values in wasps from higher exposure areas. Same metal elements were associated with decreased areas of facial melanin-based patterns in yellowjackets. These associations allowed to consider *V. vulgaris* as a biomonitor species for heavy metal pollution. We suggest the size rather than asymmetry of facial melanin-based colour patches as a morphological biomarker of metal exposure. The decrease in the area of melanin-based markings might be explained with a trade-off between physiological investment of melanin into encapsulation (detoxifying mechanism) of metal ions versus investment into colouration. Body size parameters in wasps were affected by metal loads; however, the results were not straightforward. As stated by Hodkinson and Jackson (2005), “the ideal system is inexpensive, simple, easy to implement, quick, reliable, and easily understood by non-professionals”. Our method of the assessment of phenotypic characteristics in *V. vulgaris*, the *WaspFacer*, provides a simple and convenient tool to conduct large-scale biomonitoring research using social wasps as biomonitor agents and for developing novel biomarkers for various pollutants.

## Electronic supplementary material

ESM 1 (DOCX 26 kb)

ESM 2 (DOCX 192 kb)
