# Supplementary material for: Facing the threat: common yellowjacket wasps as indicators of heavy metal pollution
Source: Environ Sci Pollut Res Int. 2020 May 18;27(23):29031–42. doi: 10.1007/s11356-020-09107-2 (PMC7376518; doi:10.1007/s11356-020-09107-2)
Supplement: Supplementary file 1 — (DOCX 26 kb) [file 11356_2020_9107_MOESM1_ESM.docx]

**Supplementary Information 1**

Wasps facing the threat: yellowjackets as indicators of heavy metal pollution

Oksana Skaldina^1^*, Robert Ciszek^2^, Sirpa Peräniemi^3^, Mikko Kolehmainen^1^,

Jouni Sorvari^1^

1 – Department of Environmental and Biological Sciences, University of Eastern Finland, PO Box 1627, FI-70211 Kuopio, Finland

2 – A. I. Virtanen Institute for Molecular Sciences, University of Eastern Finland, PO Box 1627, FI-70211 Kuopio, Finland

3 – School of Pharmacy, University of Eastern Finland, PO Box 1627, FI-70211 Kuopio, Finland

Author for correspondence: Oksana Skaldina (oksana.skaldina@uef.fi)

Supplementary tables:

**Table S1 A**. Data on the location distance, number of wasp traps and captured wasps used in the current study. Exposure zone refers to the areas closer to smelter: area 1 (≤ 2 km) and zone 2 chosen as a reference area (˃ 4 km)

| **Exposure zone** | **Distance to smelter**  **m ± SD** | **Location** | **N of traps** | **N of captured wasps** |
| --- | --- | --- | --- | --- |
| 1 | 936 ± 91 | Gate | 3 | 13 |
| 1 | 1000 ± 50 | Torttila | 5 | 154 |
| 1 | 1593 ± 0.4 | Nummi | 2 | 37 |
| 2 | 4818 ± 57 | Hiite | 5 | 12 |
| 2 | 10 592 ± 61 | Nakkila | 5 | 41 |

**Table S1 B**. Pearson correlations of the logarithmical heavy metal concentrations (µg/g) in body tissues of *V. vulgaris* (n=257) with the logarithmical distance from the Harjavalta *Cu*-*Ni* smelter

| **Element** | ***r*** | ***p*** |
| --- | --- | --- |
| *As* | -0.797 | <0.0001 |
| *Cd* | -0.606 | <0.0001 |
| *Co* | -0.407 | <0.0001 |
| *Cu* | -0.468 | <0.0001 |
| *Fe* | -0.117 | 0.061 |
| *Ni* | -0.439 | <0.0001 |
| *Pb* | -0.700 | <0.0001 |
| *Zn* | 0.155 | 0.013 |

**Table S1 C.** Pearson correlations between logarithmical concentrations of heavy metal elements (µg/g) in the body tissues of *V. vulgaris*.

|  | ***As*** | ***Cd*** | ***Co*** | ***Cu*** | ***Fe*** | ***Ni*** | ***Pb*** | ***Zn*** |
| --- | --- | --- | --- | --- | --- | --- | --- | --- |
| ***As*** |  | *r*=0.802  *p*<0.0001 | *r*=0.586  *p*<0.0001 | *r*=0.705  *p*<0.0001 | *r*=0.313  *p*<0.0001 | *r*=0.583  *p*<0.0001 | *r*=0.739  *p*<0.0001 | *r*=-0.071  *p=*0.262 |
| ***Cd*** | *r*=0.802  *p*<0.0001 |  | *r*=0.627  *p*<0.0001 | *r*=0.737  *p*<0.0001 | *r*=0.277  *p*<0.0001 | *r*=0.471  *p*<0.0001 | *r*=0.672  *p*<0.0001 | *r*=-0.019  *p=*0.754 |
| ***Co*** | *r*=0.586  *p*<0.0001 | *r*=0.627  *p*<0.0001 |  | *r*=0.661  *p*<0.0001 | *r*=0.512  *p*<0.0001 | *r*=0.733  *p*<0.0001 | *r*=0.564  *p*<0.0001 | *r*=0.325  *p*<0.001 |
| ***Cu*** | *r*=0.705  *p*<0.0001 | *r*=0.737  *p*<0.0001 | *r*=0.661  *p*<0.0001 |  | *r*=0.536  *p*<0.0001 | *r*=0.564  *p*<0.0001 | *r*=0.704  *p*<0.0001 | *r*=0.1  *p=*0.109 |
| ***Fe*** | *r*=0.313  *p*<0.0001 | *r*=0.277  *p*<0.0001 | *r*=0.512  *p*<0.0001 | *r*=0.536  *p*<0.0001 |  | *r*=0.518  *p*<0.0001 | *r*=0.315  *p*<0.0001 | *r*=0.126  *p=*0.043 |
| ***Ni*** | *r*=0.583  *p*<0.0001 | *r*=0.471  *p*<0.0001 | *r*=0.733  *p*<0.0001 | *r*=0.564  *p*<0.0001 | *r*=0.518  *p*<0.0001 |  | *r*=0.532  *p*<0.0001 | *r*=-0.047  *p=*0.447 |
| ***Pb*** | *r*=0.739  *p*<0.0001 | *r*=0.672  *p*<0.0001 | *r*=0.564  *p*<0.0001 | *r*=0.704  *p*<0.0001 | *r*=0.315  *p*<0.0001 | *r*=0.532  *p*<0.0001 |  | *r*=-0.014  *p=*0.826 |
| ***Zn*** | *r*=-0.071  *p=*0.262 | *r*=-0.019  *p=*0.754 | *r*=0.325  *p*<0.0001 | *r*=0.1  *p=*0.109 | *r*=0.126  *p=*0.043 | *r*=-0.047  *p=*0.447 | *r*=-0.014  *p=*0.826 |  |

**Table S1 D.** Pearson correlations between phenotypic parameters: melanisation area (MA), area difference (AD), Procrustes distance (PD), continuous symmetry measure (CSM) and logarithmically transformed values of heavy metals in *V. vulgaris*

|  | **MA** | **AD** | **PD** | **CSM** |
| --- | --- | --- | --- | --- |
| ***As*** | *r*=-0.367  *p*<0.0001 | *r*=-0.221  *p*=0.0004 | *r*=-0.09  *p*=0.121 | *r*=-0.108  *p=*0.085 |
| ***Cd*** | *r*=-0.221  *p* =0.0004 | *r*=-0.115  *p*=0091 | *r*=-0.035  *p*=0.582 | r=-0.061  *p*=0.334 |
| ***Co*** | r=-0.173  *p=*0.0055 | *r*=-0.115  *p=*0.067 | *r*=-0.128  *p=*0.041 | *r*=-0.125  *p*=0.045 |
| ***Cu*** | r=-0.149  *p*=0162 | *r*=-0.144  *p*=0.021 | *r*=-0.067  *p*=0.288 | *r*=-0.071  *p*=0.258 |
| ***Fe*** | *r*=-0.117  *p=*0.062 | *r*=-0.031  *p=*0.618 | *r*=-0.069  *p=*0.266 | *r*=-0.035  *p=*0.572 |
| ***Ni*** | *r*=-0.186  *p*=0.003 | *r*=-0.149  *p*=0.017 | *r*=-0.148  *p*=0.018 | r=-0.148  *p*=0.018 |
| ***Pb*** | r=-0.307  *p*<0.0001 | *r*=-0.233  *p=*0.0002 | *r*=-0.043  *p=*0.495 | *r*=-0.062  *p=*0.321 |
| ***Zn*** | *r*=0.019  *p=*0.751 | *r*=0.042  *p=*0.508 | *r*=0.019  *p=*0.762 | *r*=0.037  *p=*0.555 |
