# Supplementary material for: Facing the threat: common yellowjacket wasps as indicators of heavy metal pollution
Source: Environ Sci Pollut Res Int. 2020 May 18;27(23):29031–42. doi: 10.1007/s11356-020-09107-2 (PMC7376518; doi:10.1007/s11356-020-09107-2)
Supplement: Supplementary file 2 — (DOCX 192 kb) [file 11356_2020_9107_MOESM2_ESM.docx]

**Supplementary Information 2**

Wasps facing the threat: yellowjackets as indicators of heavy metal pollution

Oksana Skaldina^1^*, Robert Ciszek^2^, Sirpa Peräniemi^3^, Mikko Kolehmainen^1^,

Jouni Sorvari^1^

1 – Department of Environmental and Biological Sciences, University of Eastern Finland, PO Box 1627, FI-70211 Kuopio, Finland

2 – A. I. Virtanen Institute for Molecular Sciences, University of Eastern Finland, PO Box 1627, FI-70211 Kuopio, Finland

3 – School of Pharmacy, University of Eastern Finland, PO Box 1627, FI-70211 Kuopio, Finland

Author for correspondence: Oksana Skaldina (oksana.skaldina@uef.fi)

**2.5. Photographic analyses: WaspFacer program (supplementary information to the section)**

The convenient user interface (UI) of the *WaspFacer* software has been created with the purpose that several hundred photographs could be processed simultaneously and quickly. On a chosen photograph of a wasp head, a user can set an axis of symmetry in the middle of a melanin anchor-shaped facial colour marking, and manually draw a rough estimate of an area of a given colour trait. The *WaspFacer* automatically segments dark facial markings within the drawn area. Pixels within the area are grouped into three clusters utilizing the k-means++ algorithm (Arthur et al. 2007) in LAB colour space. Most of the pixels of the facial markings are contained in the cluster with the lowest mean brightness – the cluster with the darkest pixels. The pixels in the darkest cluster are defined with a user-defined value, and the largest connected component formed by the defined pixels is calculated. The largest connected component corresponds to the facial marking. Holes in the marking, which are caused, for example, by specular highlights, are filled using morphological opening operations. The threshold for the marker segmentation from the dark pixels and the extent of the marker filling can be adjusted by the user. The *WaspFacer* visualizes the contours of the segmented marking with a green contour overlaid on the original image. A user-defined number of equidistant landmarks are automatically placed along the contour on both sides of the symmetry axis (Fig. S1). We used the default value of 10 landmarks per side in our measurements.

*WaspFacer* enabled the fast and convenient assessment of several parameters for each anchor-shaped facial colour marking (Fig.2A). These parameters were: (1) a continuous symmetry measure (CSM); (2) the Procrustes distance (PD); (3) the area difference (AD); and (4) the area of melanisation (MA).

The CSM measures the mean of the squared distances that each point in the shape must be moved to transform the asymmetric shape into symmetric one (Zabrodsky et al, 1995; Liu et al., 2009)*.* If a given shape is perfectly symmetrical, the CSM value is zero. We choose to measure the CSM because facial anchor-shaped colour markings have an inconsistent symmetry with no homologous landmarks or distinct matching points (Graham et al, 2010). To calculate continuous asymmetry measure, *n* landmarks $L_{i}$ were placed equidistantly along the contour of the shape on both halves of the shape separated by the symmetry axis. The polygon resulting from the landmarks was translated to the centroid (0,0) and scaled to average centroid size. Each landmark $L_{i}$in turn was reflected along the axis to the opposite side of the axis, and the mean $\hat{L}_{i}$ of the reflected landmark $L_{i}$ and the matching landmark on the opposite side was calculated (Fig. S 2). The landmark $\hat{L}_{i}$ was then reflected the same side as $L_{i}$ and the distance $\left\| L_{i}-\hat{L}_{i} \right\|^{2}\mathrm{was}$calculated. Assuming *2n* landmarks, with *n*, corresponding landmarks for each side, CSM for a given shape was presented by the symmetry distance

$d_{s}= \frac{(\sum_{i=1}^{2n} \left\| L_{i}-\hat{L}_{i} \right\|^{2})}{n}$.

The Procrustes distance (PD) is a well-known measure of the symmetry of a given pattern (5,6). To calculate the PD between two shapes, the shapes are first “superimposed” using affine transforms (shifted, rotated, scaled). The PD is the square root of the sum of the Euclidean distances between the transformed shapes (Stegmann and Gomez 2019). However, in this context the PD was applied not for the different shapes, but for the the left and right part of the same shape, using the concept of bilateral symmetry (Mardia et al, 2000). The landmarks and the right side of the axis were reflected, scaled, translated (shifted) and rotated to minimize the distance between corresponding landmarks (Fig. S 3). To calculate Procrustes distance, landmarks on the right side of the pattern were transformed (reflected, scaled, translated, rotated) to minimize distances to points on the left side. Using the Euclidean distance between each left landmark$L_{i}$ and transformed right landmark$L_{i}^{'}$, Procrustes distance can be defined by the formula:

$$d_{s}= \sqrt{\sum_{i=1}^{n} \left\| L_{i}-L_{i}^{'} \right\|^{2}}$$

The AD was calculated as the difference in the logarithms left and ride sides of the shape. Both CSM, PD and AD have previously been applied to studying fluctuating asymmetry (FA) (4, 7). The MA is the total area of marking in mm^2^, which was estimated by using a user-defined pixels/mm ratio. Besides, *WaspFacer* enabled measurements of the distance between two points on the image, which can be utilized for measuring head width.

After the measurements had been finished, they were exported from *WaspFacer* in a comma separated format (CSV) for further analysis in SAS statistical software. The coordinates of the landmarks placed on the marking contour are exportable from *WaspFacer* in a format that is compatible with the MorphoJ software (Klingenberg 2011). However, further analyses in MorphoJ were not utilized in this study. Additionally, black and white images of the segmented markings can be exported as bitmap images (BMP) (Fig. 2B). This enables further analyses of the markings, for example, using distance transform-based methods (Taylor et al. 2013). However, this is one of the possible directions for further research. For the facilitation of research repeatability, all exported results included a metadata file. The metadata file (stored in MAT format) includes the image coordinates of the symmetry axis, markings, and automatically placed landmarks, and the settings (e.g., the threshold), that were used to produce the exported results. The metadata file can be used later to replicate and verify the performed measurements.


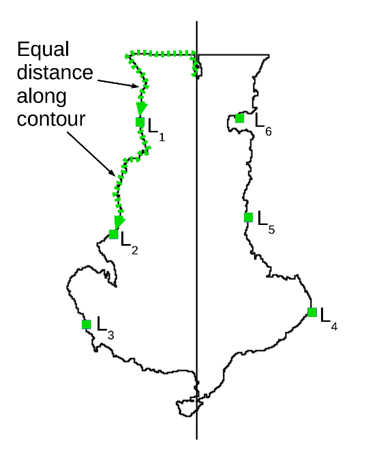


Figure S 1. Placement of landmarks on the shape contour. A total of n landmarks is placed on both sides of the shape. The distances along the contour are equal between landmarks, the start of the contour and the first landmark, and the between last landmark and the end of the contour.

**
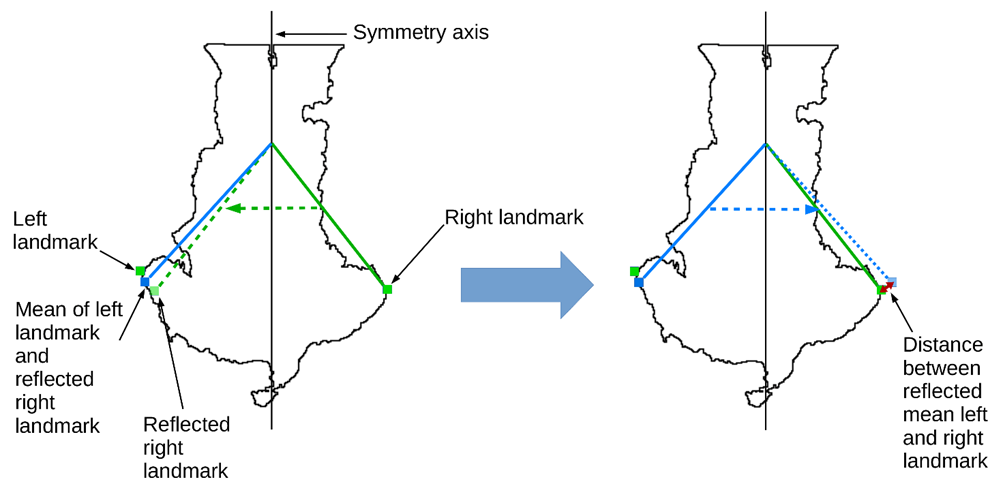
**

Figure S 2. Reflections and averaging involved in the calculation of continuous symmetry measure (CSM).


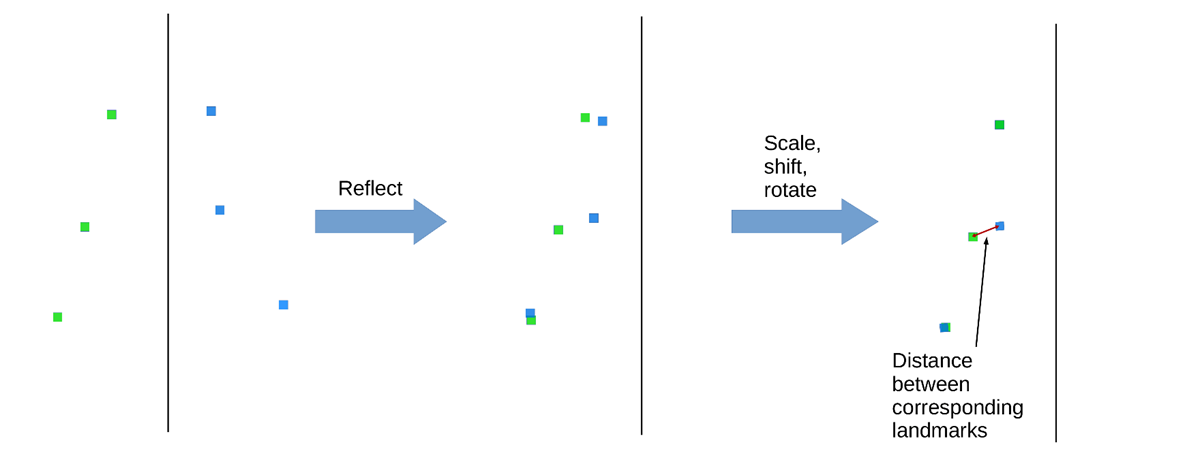


Figure S 3. Affine transforms involved in the calculation of Procrustes distance (PD). The landmarks and the right side of the axis are reflected, scaled, translated (shifted) and rotated to minimize the distance between corresponding landmarks.

**References**

1. Arthur D, Vassilvitskii S (2007) K-means. Proceedings of the 18-th annual ACM-SIAM symposium of the Society for Industrial and Applied Mathematics, January 7, 1027–1035.

2. Zabrodsky H, Peleg S, Avnir D (1995) Symmetry as a continuous feature. IEEE Trans Patt Anal Mach Intell 17 (12):1154–1166

3. Liu Y, Hel-Or H, Kaplan CS, Van Goo, L (2009) Computational symmetry in computer vision and computer graphics. Found Trend Comp Graph Vis 5(1-2):1–195

4. Graham JH, Raz S, Hel-Or H, Nevo E (2010) Fluctuating asymmetry: methods, theory, and applications. Symmetry 2 (2):466–540

5. Stegmann MB, Gomez DD (2019) A brief introduction to statistical shape analysis. Document accessed on https://graphics.stanford.edu/courses/ (Accessed Apr. 23, 2019)

6. Mardia KV, Bookstein FL, Moreton IJ (2000) Statistical assessment of bilateral symmetry of shapes. Biometrika 87(2):285–300

7. Klingenberg CP (2011) MorphoJ: an integrated software package for geometric morphometrics. Mol Ecol Res 11: 353-357

8. Beasley De AE, Bonisoli-Alquati A, Mousseau TM (2013) The use of fluctuating asymmetry as a measure of environmentally induced developmental instability: A meta-analysis. Ecol Indic 30: 218–226

9. Taylor CH, Gilbert F, Reader T (2013) Distance transform: a tool for the study of animal colour patterns. Method Ecol Evol 4:771–781
